# Everywhere and every day: the impact of navigating racialization on Black German children’s mental health

**DOI:** 10.3389/fpubh.2025.1658945

**Published:** 2025-12-16

**Authors:** Muna AnNisa Aikins, Winnie Ansah, Bridget Goosby, Laurel Raffington

**Affiliations:** 1 Max Planck Research Group Biosocial – Biology, Social Disparities, and Development, Max Planck Institute for Human Development, Berlin, Germany; 2 Department of Sociology and Population Research Center, University of Texas at Austin, Austin, TX, United States

**Keywords:** racism, internalizing, depression, children, adolescents, mental health

## Abstract

Racism is a key social determinant of health that generates racial disparities in mental and physical health, including anxiety and depression, beginning early in life. Yet, the everyday mechanisms through which racialization, including microaggressions, institutionalized, and internalized racism, affect human development are not well understood, particularly outside of the United States. This study examines how racialization influences family-level dynamics and children’s mental health and identity development in Germany, where race-evasive discourse obscures racism’s structural dimensions. We conducted six focus group discussions with *n* = 29 Black parents of *n* = 45 Black children aged 6–15 years from across the country, using a community-participatory research design. Our qualitative analysis revealed three key findings. First, systemic racism impacts family-level mobility, resources, and stress across geographic, healthcare, educational, and public contexts. Second, parents report that children experience racialization daily from early childhood (i.e., beginning at birth) with cumulative effects on mental health and identity. Third, children display behavioral adaptations to racism, such as social and academic overperformance and attempts to counter dominant racialized norms. While these strategies may offer short-term protection from social and physical harm, they may come at the cost of longer-term mental and physical health. Collectively, these findings indicate that racialization permeates the daily lives of Black German children from birth, shaping their development and mental health trajectories. Future mixed-methods and longitudinal research should examine these pathways to inform antiracist and communities-based interventions that promote children’s wellbeing.

## Introduction

Racism is a well-documented determinant of disparities in both physical and mental health (1–3). It operates through racialization—the social production and assignment of racial meaning, hierarchy, and difference in everyday life (4, 5)—a process that perpetuates inequities across structural, interpersonal, and internal levels (4, 6, 7). As a structural and institutional system of domination, racism legitimizes and sustains racialization and harms health through chronic exposure to discrimination and microaggressions, increasing the risk of anxiety, depression, and posttraumatic stress disorder (8, 9). Systemically, it also restricts access to essential social determinants of health, such as healthcare, education, housing, and employment (10, 11).

A large body of U. S.-based research highlights systemic racism as a key factor influencing the mental health of children and adolescents (12–16). Exposure to racism, both early in life and cumulatively over time, can impact stress and inflammatory systems. This, in turn, may increase vulnerability to both internalizing and externalizing disorders (1, 9, 17–19). Family contexts play a central role in how racism shapes children’s development and mental health (13, 20, 21). Parents’ own encounters with racism influence parenting practices, socioemotional development, and children’s coping strategies (22, 23).

Recent evidence indicates that similar psychosocial dynamics of racism are present in Europe (24, 25). Nearly one in four Black children in the EU report racist harassment, with the highest rates in Germany, Austria, Finland, and Ireland (26). In Germany, race-evasive discourse^1^ complicates the public and scientific recognition of racism (27–29) This contrasts with the U. S. and U. K., where racial categories are institutionally recognized and data collection on race and racial disparities is routine. As a result, racism is often rendered invisible in official statistics and social science data, where racialization is conflated with migration status (30, 31), obscuring recognition of structural inequities. While studies often focus on how discrimination and precarious conditions affect migrant mental health (32, 33), they frequently overlook the distinct impact of racialization, particularly on Black children born and raised in Germany, limiting both research and policy responses to racism.

Only a few studies have begun to address racism in Germany explicitly. Findings from the Afrozensus, Germany’s first large-scale survey on anti-Black racism—and research documenting everyday racism and resistance among youth of color further underscore the need to move beyond a migration-centered perspective and directly address the psychosocial effects of racism in Germany (34–36). Recent studies link racism to barriers in healthcare access and heightened psychological stress (37), as well as increased rates of psychosis among Black and other racially marginalized groups (38). The National Discrimination and Racism Monitor further reports higher levels of anxiety and depression among racially marginalized individuals, particularly those who are frequently subjected to discrimination (39). A significant gap remains in understanding the developmental pathways through which racialization may impact Black children’s mental health in Germany (40).

Given the absence of validated frameworks for studying racism in child development in Germany, this exploratory, community-participatory qualitative study takes an initial step toward conceptualizing racialization as a psychosocial process shaping children’s development and mental health. Conducted with a leading Black communities’ organization, the study sought to identify patterns and mechanisms from parental perspectives as epistemically grounded entry points into children’s lived experiences. We conducted six focus group discussions with 29 Black parents of 45 Black children from across Germany, addressing the following research questions:

1. How do Black parents in Germany perceive and navigate racialization in their children’s everyday lives?
2. How do they perceive links between racialization, child mental health, and development?
3. What coping strategies do children and families employ in response to racialization?

## Methods

### Study design and conceptual foundations

Grounded in anti-racist, constructivist, and community-based epistemologies, the study positioned lived experience as a source of situated knowledge (41–46). Focus group discussions (47, 48) were chosen to capture racialization as a relational and meaning-making process, enabling collective reflection on family, everyday live, and community contexts. Conducted in collaboration with Each One Teach One e. V. (EOTO)—one of Germany’s largest Black communities organizations—and the Max Planck Institute for Human Development, the study prioritized collective agency, community data governance, and the creation of safer spaces within Black community structures (41, 49). Communities partner co-developed the study design, led recruitment through trusted networks, and shared decision-making authority over data use. This participatory design recognized families as co-producers of knowledge and sought to produce findings that are scientifically rigorous and socially accountable to Black communities in Germany.

### Participants and recruitment

Participants were 29 self-identified Black, African, or Afro-diasporic parents (19 mothers, 10 fathers) in total raising 64 Black children, of whom 45 children aged 6–15 fit the analytic focus. This age range represents a critical developmental period when racial awareness, identity formation, and mental health vulnerability become particularly salient (13, 17, 50, 51). Recruitment followed an anti-racist and empowering approach, prioritizing informed consent, agency, and collective participation. Participants were recruited through EOTO using purposive and snowball sampling via communities’ networks, social media, and outreach platforms. Collaboration with EOTO ensured trust, cultural safety, and accessibility, enabling open discussion of racism—topics rarely addressed in institutional research contexts in Germany.

A total of six online focus groups were conducted via Zoom, each lasting approximately 90 min and comprising three to six participants. Each parent received 50 Euro compensation for participation. Groups varied in their composition with respect to parent gender, child age, and occupational background. More mothers participated than fathers, with roughly half of the groups being balanced and the other predominantly composed of mothers. Although the initial design aimed to stratify groups by children’s ages, this was not feasible because many parents had children across multiple age ranges. As a result, discussions often included references to siblings of different ages; however, the accounts referring to children aged 6–15 years were analyzed to maintain the study’s focus. The majority of participating families lived in major urban centers, including Berlin, Hamburg, Munich, Frankfurt, Freiburg, Hannover, and Saarbrücken, while a few were located in mid-sized towns or rural regions. To protect confidentiality and reduce the risk of identification from combined descriptors (e.g., children’s ages, gender, residence, and parents’ professions), we report only broad residential categories: large city, medium-sized city, small town or village, or rural area. A detailed demographic overview is provided in Supplementary material 2.

### Data collection procedures

Data were collected using a semi-structured focus group discussion guide with open-ended questions and follow-up prompts (47, 48) to facilitate in-depth engagement (see Supplementary material 1). The guide contained prompts exploring parents’ perceptions of racialization, children’s psychological and behavioral responses, and coping resources. Questions prompted parents to reflect on moments when children felt different because of race, skin color, or hair, and how these experiences related to their mental health and sense of self. Example prompts included:

- “Can you share your experiences as a parent raising a Black child?”
- “Can you recall a time your child felt different because of race, skin color, or hair?”
- “In your view, are these experiences related to your child’s health or well-being?”

Each parent participated in one focus group. All focus groups were moderated by the same facilitator, a Black academic and parent of Black children, to minimize power asymmetries and to support open, trust-based dialogue. Recognizing that racism is often silenced within German institutions, the research team worked closely with EOTO to co-create a safer research space. Throughout data collection and analysis, the team practiced cultural humility, understood as an ongoing commitment to reflexivity, self-critique, and the intentional addressing of power imbalances in community-based research (52). All sessions were audio-recorded with informed consent and professionally transcribed. Transcripts were anonymized, with all personal identifiers removed, and checked for accuracy. Four sessions were conducted in German and two in English. German excerpts used in the manuscript were translated into English using a meaning-oriented approach (see Supplementary material 7).

### Data analysis

The analytic process was guided by situated knowledge and feminist standpoint epistemology, emphasizing reflexivity, transparency, and accountability to community perspectives (43, 45, 46). Research team members regularly reflected on how their social positions, including race, gender, and professional status, shaped interpretation and coding. This reflexive stance helped ensure that the analysis remained grounded in participants’ lived experiences of racialization and attentive to the power dynamics inherent in community-based research, consistent with principles of cultural humility (52).

Following an inductive thematic approach (53), transcripts were repeatedly read to identify initial concepts and contextual nuances. Two researchers jointly coded an anchor transcript to develop the initial codebook, refining it through analytic memos and discussion. Three focus groups (≈50% of the dataset) were then independently double-coded to assess interpretive alignment. Intercoder agreement was calculated using MAXQDA’s code-overlap function with a strict 85% overlap threshold and the “segments of both coders” option (54, 55). Across 27 thematic codes, mean observed agreement was 71% (Cohen’s *κ* = 0.69), indicating substantial agreement. Detailed results, including per-code agreement levels are provided in Supplementary material 4.

Coding discrepancies were resolved through discussion, refining code definitions and strengthening shared interpretation. Consistent with grounded-theory-informed practices, intercoder agreement is reported for transparency rather than as a validation metric; divergent coding was treated as analytically productive. Themes were then clustered into higher-order categories, resulting in five overarching thematic domains (see Supplementary materials 5, 6).

A final integrative synthesis links data-derived themes (e.g., systemic racism, internalized stress, coping) with theoretically informed models of race-critical developmental pathways (13, 17, 20). This synthesis presents a conceptual model connecting racialization processes to children’s mental health trajectories and outlines hypothesized pathways for future longitudinal and mixed-methods research.

**Figure 1:**
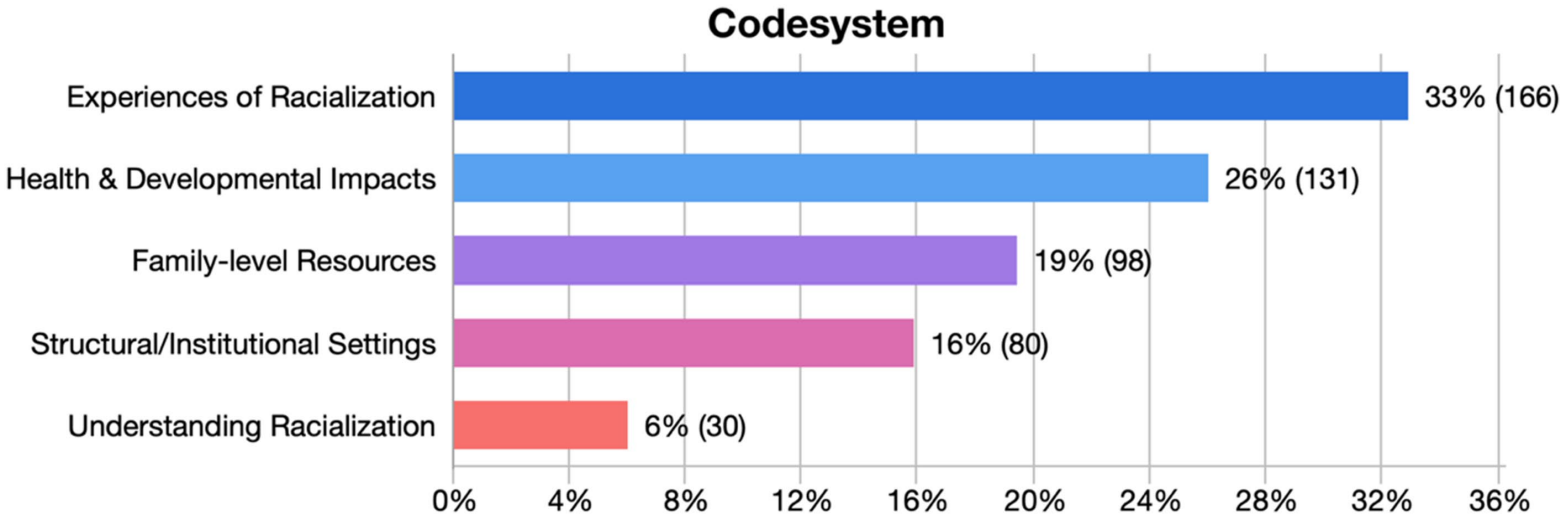
Relative frequencies of thematic categories reported by parents. The figure presents the distribution of coded segments (*N* = 505) across the overarching themes identified through inductive analysis. Percentages indicate the relative proportion of coded segments within the full dataset, and the numbers in parentheses show the count of coded segments per category.

**Figure 2:**
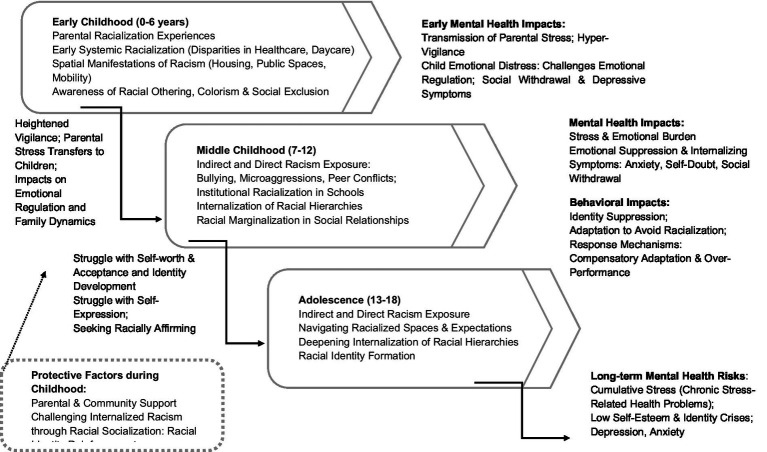
Conceptual model of hypothesized developmental pathways linking systemic racialization and child and adolescent mental health. This figure integrates the preliminary qualitative insights of this study with race-critical developmental frameworks to illustrate how systemic racialization, internalized racism, and cumulative stress may interact across developmental stages—early childhood (0–6), middle childhood (7–12), and adolescence (13–18)—to shape mental health trajectories within structural and familial contexts.

### Methodological integrity and reflexivity

Methodological integrity (56) was maintained by balancing fidelity to participants’ narratives with the study’s developmental and race-critical aims. The interdisciplinary research team comprised four scholars—three Black (including the lead author) and one white—trained in developmental psychology, sociology, and community-based research. Led by Black scholars with lived experience of racialization in Germany, the study’s design and interpretation were grounded in ethical engagement and accountability to community perspectives. Guided by principles of cultural humility (52) and standpoint theory (43, 45, 57), the team engaged in ongoing reflexive practice through memo writing, analytic debriefings, and positionality discussions, critically examining how racial, gendered, and institutional positions shaped interpretation. Triangulation across six focus groups, reflexive dialogue among coders, and bilingual (German/English) analysis enhanced interpretive depth, and divergent readings were resolved collaboratively, privileging meanings grounded in participants’ own words.

### Ethical framework and approval

The study followed strict ethical protocols, emphasizing informed consent, transparency, and participant empowerment. Information sessions were held before and after focus group participation to allow questions and clarify study details. Ongoing communication channels were established to maintain participant engagement. Ethical approval was granted by the Ethics Committee of the Max Planck Institute for Human Development.

## Results

The inductive thematic analysis generated several overarching thematic categories capturing how parents described racialization and its developmental and mental health implications for their children. As shown in Figure 1, these categories encompass experiences of racialization, health and developmental impacts, family-level resources and coping, structural and institutional settings, and parents’ understanding and anticipation of racialization. Together, these categories comprised 27 subthemes and 505 coded segments (see Supplementary material 5 for the full codebook with codes and their definitions, and Supplementary material 6 for top-level and subcodes structure and frequencies). Frequencies are reported to illustrate relative narrative emphasis, providing a transparent overview of topic salience across the dataset.

Building on this structure, the following sections present parental perspectives on racialization and child mental health, organized around three central themes synthesized during analysis:

First, impact of systemic racism on family dynamics and access to resources. Second, impacts of racialization on children’s mental health and identity formation. Third, child’s behavioral adaptation such as attempts at overperformance in response to racialization.

### Theme 1: impact of systemic racism on black families

Black parents reported that the early onset of racialization began at birth, particularly in medical settings where healthcare professionals’ comments and treatment reflect racialized assumptions about skin tone and hair texture. They described disparities in access and quality of care highlighting how racism permeates the healthcare system, shaping children’s earliest experiences.

*“I would almost like to start with the birth experience, as an object of racism or racialization. Showing up at the hospital as a black family makes so that's where it starts, right? And also, the comments after the birth. ‘How dark is it going to be?’ And: ‘Oh, the hair looks”, I don't have to reproduce everything, you know. But I would start, (laughs) quite honestly, with the medical treatment and all those first comments. Difficult. So, the process is noticeable right from the start. Just like that, racialization.” (Mother of two daughters, ages 4 and 8; Focus Group 1, pos. 22, translated)*

As children grew, parents observed recurring overt racism, microaggressions, and racial othering in daycares, playgrounds, and extended white family settings. These experiences prompted ongoing family discussions on racism, as parents sought to equip their children with coping strategies.

Parents described how the spatial manifestations of racism shaped their experiences in housing and public spaces, limiting opportunities through discrimination and ongoing safety concerns. Racialization influenced decisions around housing, neighborhood, and mobility. Experiencing heightened surveillance, over-policing, and restricted access further constrained their movement, with fears of racial hostility prompting avoidance of certain areas and increasing parental vigilance to protect their children.

*“These restrictions in everyday life are there every day, every day. I have, so I - for me it's a constant film, always checking where we are. Where do we get on the tube when we're travelling? And to make sure that we have as few such experiences as possible or that I try to avoid having them. Of course, I can't always avoid it and control everything, but I can take preventative measures to ensure that the risk is really minimized.” (Mother of a daughter, age 10; Focus Group 1, pos. 34, translated)*

Parents described how structural racism limited access to rural and suburban areas, forcing their families into smaller, costly urban zones. While cities offered more diversity and relative safety, they came with trade-offs like reduced space and limited access to nature. In rural areas, parents reported social isolation, racial othering, and hyper-critical neighbors, reinforcing structural barriers and deepening housing inequities.

*“Then I keep realizing how incredible it is that I can't live where I actually want to live with my children. I think that's really tough. …. And we live in the center forever.” (Mother of a daughter, age 10; Focus Group 1, pos. 29, translated)*

Parents described how institutional racialization unfolded in schools and kindergartens—key sites of both structural and interpersonal racism—where their children encountered verbal insults, discriminatory treatment, and lowered academic expectations. They expressed deep concern about unequal treatment based on skin color, highlighting the constant need for vigilance and advocacy within educational settings.

They emphasized how systemic racism and family mental health are intertwined, as stressors such as economic hardship, discrimination, and time constraints affect both their mental health and that of their children. These pressures hinder parents’ ability to provide emotional support and model regulation, often leading to premature self-reliance in children, with lasting impacts on their mental health and cognitive development.

*“I'm also thinking about attention, like space and energy and time to be present with our kids and how that is minimized because we have to hustle. Maybe we have to work longer times or we are stressed because we are also within these intersections and experiencing systemic, based stress, and that takes away our capacity to be present and to regulate and to empower and strengthen our children. That also has an effect in the sense of some processes in the brain or developmental processes, which means that our children have to figure out things earlier by themselves and that also creates stress again.” (Mother of a daughter, age 7; Focus Group 2, Pos. 49)*

The constant anticipation and management of racialization took a toll on parental mental health, reinforcing intergenerational and cumulative stress. This emotional strain impacted parenting, as parents navigated both their own and their children’s racialized experiences.

*“Where I'm already prepared that it's critical to present myself one way or another. And that's more my experience, that it's more stressful for me. I have a high stress level, always on guard. (…) Because I don't want to expose him to that* [referring to racist incidents] *if I can avoid it.” (Mother of a son, age 6; Focus Group 6, pos. 22, translated)*

[Quote is part of a longer discussion of racialized neighborhood dynamics]: “*I also feel that as parents we have a lot of stress. (…) The moment you enter your house; it's supposed to be a place where you feel calm. However, then you also have to deal with nosy neighbors, angry neighbors, all these things. I feel like also that stress transfers to the kids as well. There's no way around it.” (Mother of a daughter, age 7; Focus Group 3, pos. 107)*

### Theme 2: impacts of racialization on children’s mental health

Parents described how microaggressions, stress, and mental health are closely connected in their children’s lives. Racially marginalized children regularly face both overt racism and subtle microaggressions that shape their identity and health. These experiences create chronic stress, often forcing children to navigate racialized realities beyond their developmental capacity. Comments targeting physical traits frequently trigger emotional distress, leading to struggles with self-acceptance, social withdrawal, depression, and emotional exhaustion.

*“Now he's at school, the big one, and there's already the first comment that comes out about the color of his skin: ‘You're as stupid as the color of your skin.’ (…) He has good reflexes, but he still doesn't like his hair. He would like to have it straight. And, right, now he accepts his skin color. That was also an issue. So, until he had childhood depression like that, he didn't want to go anywhere.” (Father of a son, age 7; Focus Group 5, pos. 88, translated)*

*“She cries every day because you feel like most kids have beautiful hair and long hair, and she doesn't have this kind of hair.” (Mother of a daughter, age 12; Focus Group 3, Pos. 88)*

Many children internalize racialized experiences rather than openly discussing them. Some cope by suppressing their emotions and needs, making it difficult for caregivers to recognize their struggles.

*“But I think that's how they, he experiences more, so my son now. And just doesn't talk about it so much then.“(Mother of a son, age 11; Focus Group 5, pos. 98, translated).*

Parents expressed concern about the emotional toll and long-term impact of internalized racism on their children. They observed how early exposure to racial hierarchies—especially colorism—shaped children’s self-perception, behavior, and emotional responses. Navigating racial othering, exclusion, and peer conflict often required children to assert boundaries while managing distress. Difficulties with self-expression, particularly in responding to microaggressions or voicing opinions, were common, highlighting the deep effects of internalized racism on identity and mental health.

[Mother quoting her son]: *“I only have one friend who sometimes doesn't want to play with me either.’ And: ‘I'm different. Maybe they don't like me because I'm different.” (Mother of a son, age 8; Focus Group 4, pos. 41, translated)*

Eurocentric beauty standards significantly influenced children’s self-worth, particularly regarding hair and skin tone. Parents observed instances where children favored lighter-skinned characters in books or avoided wearing brown clothing out of fear of appearing “too brown.”

*“The middle child wanted, had also problems with the color brown. So, he didn't want to wear his brown trousers. Exactly, we went through that. We knew what was happening, what, why does he suddenly not want to wear brown clothes? Yes, because he doesn't want to appear any browner” (Mother of a son, age 6; Focus Group 5, pos. 92, translated)*

Some parents observed that Black children, influenced by colorism, sometimes projected rejection onto peers of the same racial background, highlighting the deep impact of internalized racism.

*“And when it comes to internalizing, I can't help but think of something else that is very shocking and very intense for me at school: racism between Black children. Because I have the impression that a lot of internalized hatred and rejection is projected by some children onto other Black children.” (Mother of a daughter, age 8; Focus Group 1, pos. 24, translated)*

Parents noted that identity formation is shaped by the struggle for belonging, as children navigate marginalizing societal structures. Racial othering and repeated racialized experiences often undermine their self-esteem and sense of belonging.

*“He might not feel that he belongs to the society in which he actually lives.” (Mother of a son, age 8; Focus Group 6, pos. 28, translated)*

They emphasized the long-term risks of identity suppression, explaining that children may internalize the belief that conforming to dominant norms minimizes racialized exclusion.

*“Because of this identity development, they have a harder time finding out who I actually am? Because they have so often simply learned that it works or (…) less happens to me if I simply behave like the others. (…) And I think that's a danger that they lose that and then later simply experience such a blatant identity crisis.” (Mother of a son, age 8; Focus Group 6, pos. 35, translated)*

Parents stressed that affirming children’s natural hair and racial identity is crucial in countering the effects of racialization. Positive reinforcement strengthens self-perception and confidence when navigating racialized interactions, such as unwanted physical contact or exclusion. This support is essential for their children’s mental health.

*“She was feeling not enough because she was maybe the only one or the only girl with this kind of hair. You have to put in more effort to talk to your kids about what makes them special and why she's so beautiful the way she is. Finally, after some time, she learned to accept herself and is even now proud of her hair.” (Mother of a daughter, age 10; Focus Group 3, pos. 85)*

### Theme 3: children’s behavioral adaptations to racialization

Parents noted that adapting to Eurocentric standards shaped how their children navigated social spaces. In response to racial hierarchies and the pressure to avoid othering, children often modified their behavior and appearance—strategically managing visibility while constraining self-expression.

*“She has now changed schools (…) so we are ten, she is actually so far along that she understands that with straight hair, that it brings privileges (…) then she actually said: ‘Mum, I'm going with an afro. I'm going with an afro because then I'll have my peace. They see my hair as it is once (…). And then they don't ask any stupid questions.” (Mother of a daughter, age 10; Focus Group 1, pos. 56, translated)*

Daily microaggressions reinforce the need for constant compensatory adaptation, causing some children to suppress emotions, prioritize others’ needs over their own, and avoid conflict.

*“Well, a lot of adaptation. So always looking at what the others need, what do the others want? What do they want from me? What do they expect from me? I do that because then I'm more confident. That alone is of course a kind of stress factor.” (Mother of a son, age 13; Focus Group 1, pos. 56, translated)*

*“It's this over-adaptation; I think someone mentioned it earlier. (…) That in situations that really shouldn't be like that, he learns that he has to be the one who always has to keep his mouth shut, who always has to keep a low profile.” (Mother of a son, age 8; Focus Group 6, pos. 35, translated)*

Parents highlighted the mental health toll of constant adjustment to avoid negative attention, warning that compensatory adaptation could lead to identity struggles or crises, especially during adolescence.

They observed that attempts at academic overperformance was a common coping strategy among their children, who responded to societal pressures by striving for perfection and heightened compliance. To avoid racial marginalization, children often felt compelled to exceed expectations both academically and socially, aiming to minimize the risk of standing out as Black children.

*“I also see a blatant perfectionism in her, for example. She wants to do everything particularly well, so she doesn't want to stand out with negative results or achievements. And the stress that this causes, no, to be good all the time, to be right, yes, to be decent and so on.” (Mother of a daughter, age 10; Focus Group 1, pos. 56, translated)*

[Mother recalls her 11-year-old son saying]:‘*I make sure that I have very high, very high expectations of myself. I just want to be very good. Very if it's not done well at first, then that frustrates me. I just want to be very, very good.’-* [continues]: *“And I think that also has to do with his skin color. Just the, to shine where he can shine, because he, yes. Because he has realized that being different can also be a disadvantage.” (Mother of a son, age 11; Focus Group 5, pos. 103, translated)*

To cope with racial marginalization, children often form friendships with peers who share similar racial and social backgrounds. Parents observed that these connections offer comfort, emotional security, and a sense of belonging, which support children’s responses to racialization.

*“And now, by chance, another Black boy is joining his class (…) he's now the only Black boy in the class. He just said yesterday that he's happy that he's no longer the only one. (…) they feel more comfortable when they can identify themselves” (Mother of a son, age 6; Focus Group 6, pos. 31, translated)*

*“The positives are that she picks her friends very carefully and with reason. She wants to be with this person because they make her feel happy and play with and she doesn't want to play with this person because he keeps touching her hair.” (Mother of a daughter, age 7; Focus Group 2, pos. 36)*

As children transition into adolescence, the search for belonging becomes more deliberate. Teens actively seeking out peers who share similar racial or social experiences, forming relationships that reinforce their identities. These connections provide a sense of community and play a crucial role in supporting mental health, especially in the face of racialized experiences.

*“My 14-year-old daughter, that she's already changing her circle of friends a bit at her age. (…) she's getting more involved in the Black community. Not that her previous circle of friends is bad or anything. But I think she realizes that she can identify with it a bit better now, even at that age. And perhaps also feels more comfortable and they have the same topics and so on.” (Mother of a daughter, age 14; Focus Group 6, pos. 31, translated)*

Across themes, parents described racialization as pervasive and cumulative, shaping family stress, children’s mental health, and identity from early childhood through adolescence. They also emphasized the protective potential of racial socialization and community networks. Figure 2 presents a conceptual model of hypothesized developmental pathways linking racialization and child mental health. The model integrates data-derived themes with established race-critical developmental and ecosocial frameworks (13, 14, 20, 21, 58), outlining how systemic racialization, internalized racism, and cumulative stress may interact across developmental stages to shape mental health trajectories. These pathways merit further investigation through longitudinal and mixed-methods research.

## Discussion

This community-partnered qualitative study examined racialization experiences and how they shape family dynamics, children’s mental health, and identity development in Germany. Three interrelated themes emerged from parents’ accounts. First, parents described how systemic racism shapes family mobility, access to resources, and exposure to stress across geographic, healthcare, educational, and public contexts. Second, parents observed that children encounter racialization from early childhood, which contributed to internalized racism and psychological strain, including emotional distress, social withdrawal, and identity challenges. Third, children appeared to adapt behaviorally to racism through efforts to socially or academically overperform in order to counter dominant racialized norms.

The first theme was reflected in parents’ accounts of restricted mobility, surveillance, and discriminatory treatment across public and institutional spaces. We found that parents perceived racism in Germany as substantially shaping their families’ access to resources across public spaces, contributing to family stress. Many described heightened vigilance and safety concerns, often choosing urban environments for relative security despite trade-offs in quality of life. Reports of housing discrimination, experiences of othering, and over-policing in public spaces were common and constrained families’ mobility, potentially reinforcing racial segregation. These marginalization dynamics echo findings from the U. S., where race-based residential segregation perpetuates disparities in health and wellbeing through both social and environmental exposures to toxic stressors (11, 59–61). Parents in our study observed that racism is a constant in everyday life and that the racialization of Black children begins at birth.

Parents emphasized that racial hierarchies shape unequal treatment in schools, and requires their continued advocacy within the education system. They described schools as central sites, where racial hierarchies become institutionalized, noting that Black children are frequently subjected to marginalization, verbal assaults, and the devaluation of their academic potential. Such experiences were perceived as contributing to stress, lower self-esteem, and long-term mental health struggles (62). These accounts mirror broader racial disparities: EU-wide, 23% of Black parents report that their child has faced racist comments (26). In Germany, that share rises to 38%, with 13% reporting their child experienced 18% social exclusion, physical abuse and 18% social exclusion.

Moreover, parents described how the intersection of racialization and economic precarity intensified family stress and created additional mental health risks for both children and caregivers. They noted that systemic racism limits access to economic and social capital, which in turn restricts parental investment in education and healthcare. This lack of resources can hinder emotional support, reinforce vigilance and avoidance behaviors, and can limit children’s social experiences, ultimately undermining their long-term health and development (63–65).

Persistent safety concerns in public spaces added to families’ cumulative stress. U. S.-based research links repeated exposure to poverty, discrimination, and social exclusion with chronic stress and long-term health impairments (21, 66). Increased parental stress can lead to children’s emotional dysregulation and behavioral difficulties (67, 68). Structural inequities, including housing instability, wage gaps, and unequal access to services, further intensify cumulative stress and perpetuate intergenerational disparities (10, 69). Our findings indicate that similar mechanisms may operate in Germany, where structural racism is deeply embedded in institutions and everyday life (34, 39).

The second theme focused on the impact of internalized racism, shaped by racial hierarchies and colorism, on children’s developing identity and mental health. Parents noted that from an early age, children became aware of racialized differences, influencing their behavior and self-worth. Eurocentric beauty standards often played a central role in this process, often leading children to reject darker skin tones and internalize racial narratives. These observations align with prior research linking internalized racism to increased psychological stress and diminished self-esteem (70–73).

The third theme captured children’s behavioral adaptations to racism. Parents noted that their children adopted coping strategies such as compensatory adaptation, perfectionism, and identity suppression to navigate racialized contexts. These avoidance-based coping mechanisms may provide short-term protection but can also lead to emotional suppression, deeper internalized racism, and longer-term mental and physical health costs may offer short-term relief but can result in emotional suppression and further internalized racism (74–76). Overperformance, particularly in academic and social settings, often functioned as a strategy to counter racial narratives and affirm self-worth. This behavior aligns with John Henryism, a detrimental coping strategy observed in Black communities in the U. S., characterized by relentless overexertion for acceptance, which incurs long-term psychological costs such as stress and burnout (77–79).

Racialization was described as profoundly shaping Black children’s mental health, shaping identity, self-esteem, and social relationships from early childhood. Parents reported that overt racism and daily microaggressions often contributed to emotional distress, self-doubt, social withdrawal, and identity struggles. Studies consistently link racialized stressors to increased anxiety and depression (16), and to disrupted socioemotional development (17). Parents in this study observed that repeated exposure to racialization reinforced internalizing symptoms and internalized racism, generating cumulative stress and posing long-term mental health risks.

Quantitative longitudinal research from the U. S. shows that racial disparities in internalizing and externalizing behaviors are already observable in early childhood (1) and that racial discrimination during adolescence predicts mental health deterioration into adulthood (80) Moreover, racialized differences in adolescent mental health are associated with increasing racial disparities in biological aging, as measured by epigenetic clocks (1). In adults, epigenetic-clock measured biological aging partially accounts for racialized health disparities (81). Thus, early racialization may contribute to enduring health inequities across the life course, as racial disparities in multimorbidity and lifespan are closely tied to patterns of biological aging.

In our focus groups, parents emphasized the importance of racial identity in bolstering their children’s abilities to cope with racism. Black children often seek out relationships that affirm their identity and avoid spaces where they face racial marginalization. Thus, racial socialization with similarly affected peers provides opportunities to mitigate the psychological effects of racism (13, 20, 50, 82). Accordingly, a strong racial identity has been associated with fewer internalizing symptoms (18). Social support, especially through peer networks and racially affirming communities, appeared to enhances coping capacities. However, parents also indicated that access to such spaces is often limited by racism (83, 84).

## Conclusion

Drawing on the perspectives of Black parents, this study shows that racism in Germany operates as an ongoing stressor that shapes the everyday experiences, mental health, and identity development of Black children. As an exploratory, grounded-theory–informed study, it offers an initial foundation for understanding how racialization may affect children’s developmental trajectories. Future research should include children’s perspectives and broader sociodemographic indicators to investigate how racialization intersects with other structural inequities. Mixed-methods, longitudinal, community-based, and child-centered approaches, including ecological momentary assessment, passive stress tracking, and participatory methods, are crucial for capturing lived experiences without reproducing racialization processes. Our findings also highlight areas where anti-racist policy and practice are needed: mental health services need to adopt racism-responsive, trauma-informed approaches, and municipalities should strengthen Black-led organizations and expand access to safe, affirming spaces. Finally, research and data governance in Germany must move beyond race-evasive frameworks toward ethical data-justice practices that enable the documentation and reduction of racial inequities.

## Data Availability

The datasets generated and analyzed during the current study are not publicly available due to legal, ethical, and privacy restrictions. Reasonable Requests to access the dataset should be directed to organization Each One Teach One eV. Requests to access the datasets should be directed to info@eoto-archiv.de.

## Footnotes

1 Race evasiveness refers to the intentional avoidance of engagement with race, racism, or racialization. It is not merely a lack of awareness; it is a strategy that maintains dominant racial hierarchies by rendering racism invisible. In the German context, it manifests through the framing of racism as a historical or foreign problem, and the corresponding failure to acknowledge it as a persistent, structural feature of society [see (27)].
